# Embodied contradictions, structural power: Patient organizers in the movement for global health justice

**DOI:** 10.1371/journal.pgph.0002577

**Published:** 2023-11-07

**Authors:** Apoorva Gomber, Eunice Owino, Moses Echodu, Anu Gomanju, Paladie Mategeko, Lauren Brown, Jonathan D. Shaffer

**Affiliations:** 1 Center for Integration Science in Global Health Equity, Brigham and Women’s Hospital, Boston, MA, United States of America; 2 NCDI Poverty Network, Boston, MA, United States of America; 3 Sickle Cell Uhuru Trust, Kenya (SCUT), Nairobi, Kenya; 4 Uganda Child Cancer Foundation, Kampala, Uganda; 5 Kathmandu Institute of Child Health (KIOCH), Kathmandu, Nepal; 6 Rwanda NCD Alliance, Kigali, Rwanda; 7 Partners in Health, Boston, MA, United States of America; 8 Department of Sociology, University of Vermont, Burlington, VT, United States of America; PLOS: Public Library of Science, UNITED STATES; McGill University, CANADA

On June 13th, a boat full of people seeking refuge in Italy capsized off the coast of Greece, mustering little if any effort from European authorities to support, rescue, or care. More than 700 men, women, and children perished [1]. A day later, the world jumped into motion to try to save, and then attempt to recover, the five wealthy people from a misguided submarine adventure to visit the ruins of the Titanic. A four-nation, multi-million-dollar response and investigation effort was able to recover the wreckage and remains of the ill-fated divers [2].

Juxtaposing the media attention, public response, and governmental action of these two tragedies and the global forces that created them point to a glaring, structuring reality within the forces that govern ‘global health’ as well. Whose life is valued and worth caring for? Whose life is seen as a burden and thus expendable? These questions are not new. From the struggle to expand access to anti-retroviral medication to the recent Covid-19 vaccine inequities, global health has long tradition of under-valuing the lives of Indigenous, Black, and brown people, especially in the Global South [3].

The current regime of global health governance ensures three things remain true.

First, it ensures that the capital allocated to systems of effective caregiving remains deeply insufficient for the health needs of the vast majority of the people in the world [4]. Indeed, at least half the world lack access to essential health services [5]. Second, the fragmented caregiving systems that do exist are largely governed by global institutional philanthropy and their NGOs, usually from the U.S. or Europe, and deploy a logic of “cost-effectiveness”–a race to the bottom in terms of care quality in the name of “efficiency” [6]. Third, this regime of fragmented charity care in impoverished regions of the Global South often dampens the political aspirations of patients, healthcare providers, and Ministry of Health planners. Sights get narrowed; expectations are reined in because budgets are assumed to be fixed. Failures of imagination combine with deep socialization for scarcity such that substantive change feels insurmountable.

Attempts to transform this unjust global health system have generally failed. Aspirations to expand conceptions of universal access to robust primary health care advanced during the WHO’s meeting in Almaty, Kazakhstan in 1972 were quickly rolled back with “more realistic” calls for “selective primary health care” [7]. The movement in the 1980s and 90s to expand access and systems to deliver effective treatments for HIV/AIDS has been touted as perhaps global health equity’s greatest success story. But that “success” has nonetheless left nearly 10 million people without access to necessary, life-saving antiretroviral therapy today. For far too many, HIV/AIDS is still a life-or-death struggle while the tools and know-how exist [8].

Where do we find hope given this bleak picture?

We, the authors, find hope in the fights waged by our fellow patient-organizers. Patient organizers are people living with disease, and those who stand in solidarity with them, who choose to build organizing campaigns and power within a broader constituency to win shared goals. They are central in driving what sociologists have called *embodied health movements* [9].

We find it in the embodied contradictions represented by patient organizers around the world who know what’s at stake and demand more; the true subject experts who recognize the unique challenges they and their communities face [10]. Hope springs from the network of patient-organizers who are actively reframing non-communicable diseases and injuries (NCDIs) among the poorest billion people and who are launching local social movement organizations focused on the profound gaps in care and services available to people living with severe noncommunicable diseases (NCDs) [11].

The *NCDI Poverty Network*, which was established in 2020, has now expanded to include national commissions from 22 lower-income countries. The network aims to broaden the NCD agenda in the interest of equity and find solutions to highly constrained health systems through integrated using shared infrastructure. This network is technically and politically struggling to address severe chronic NCDs—such as type 1 diabetes, sickle cell disease, congenital and rheumatic heart disease by implementing *PEN-Plus*, an integrated care-delivery model for chronic care services at first-level rural hospitals [12]. The NCDI Poverty network encourages patient-organizers to fight for a more prominent voice in regional and global dialogues around NCDs and injuries. It has spurred a new movement of patient advocacy through an initiative called ‘Voices for PEN-Plus’, which is expanding conceptions of global solidarity and activism throughout the Global South [12].

Working as and alongside these organizers has taught us important lessons about what’s needed to scale movements for health justice.

First, building interdependent teams capable of building solidarity, recruiting more leaders and organizers, and developing increasingly sophisticated campaigns is crucial in growing movements for health justice. It’s a difficult task, but we have found significant value in learning alongside serious organizers from health justice movements from around the world through engaging with the *Leading Change Network’s Health Justice Hub* [13]–a growing community of dedicated organizer-trainers who work together to win campaigns, learn organizing skills, grow their movement organizations.

Second, learning the craft of telling public stories about encountering personal and public challenges, but also finding the courage and resilience to challenge them and fight back, is crucial to building the emotional capacity necessary to struggle for change [14]. Here, patient organizers are especially powerful–they have encountered profound biosocial challenges in their lives, and yet have made agentic choices to work with others and organize within their constituency to fight for services and care for themselves and their community.

Third, and most importantly, patient-organizers work to develop shared strategies for change–working theories about how they can use the resources of their people to force changes that can solve the complex problems they face. Global health practice extends into the intersections of health, social, political, and non-health sectoral issues bringing multi-disciplinarity in global health expertise. It’s indeed essential to recognize that people with lived experiences have a crucial perspective to offer when it comes to creating policies and programs to address the issues they have faced.

Unfortunately, the progress of building a movement is impeded by insufficient resources, funding, and informal methods for enhancing skills and abilities. Many policymakers, charitable donors, and decision-makers with considerable power are hesitant to support grassroots movement organizations which are often viewed as a potential political liability, imagined to be disorganized or ineffective, or otherwise downplayed as mere ‘lobbying’. The funding that does exist is often tokenistic and does little to nothing to enable these patient organizers to grow their own capacities and power within their constituencies. These funders generate a culture of neoliberal individualization encouraging competition rather than collaboration, which can result in creating silos [15].

We firmly believe that community organizing efforts are crucial to transform global health but are still grossly undervalued and overlooked. However, the organizing work currently underway with Voices for PEN-Plus gives us hope and evidence that we can forge strategies for global solidarity and collective action. This can pave the way for promoting health justice worldwide by dismantling oppressive systems and ensuring that everyone has access to healthcare as a basic human right and is the cornerstone of health equity.
